# Traditional Chinese Medicine Bone‐Setting Techniques Research Progress for the Treatment of Knee Osteoarthritis

**DOI:** 10.1155/bmri/9073869

**Published:** 2026-06-27

**Authors:** Qiao Fan, Ming-Yu Zhao, Xiang-Dong Zhang, Tian-Yun Chu, Zhao-Xi Kou, Qi Zhao, Nan Wang, Xiao-Ying Liu, Jia-Hao Zhang, Qiu-Sheng Wang

**Affiliations:** ^1^ Henan Luoyang Orthopedic Hospital (Henan Provincial Orthopedic Hospital), Zhengzhou, Henan Province, China; ^2^ Graduate School, Hunan University of Chinese Medicine, Changsha, Hunan Province, China, hnctcm.edu.cn; ^3^ Graduate School, Henan University of Chinese Medicine, Zhengzhou, Henan Province, China, hactcm.edu.cn

**Keywords:** knee osteoarthritis, summarize, traditional Chinese medicine bone-setting techniques

## Abstract

This paper systematically reviews the biological mechanisms and clinical applications of traditional Chinese medicine (TCM) manual therapy in the treatment of knee osteoarthritis (KOA), explores the scientific basis for its multipathway intervention in improving joint function, and analyzes the current research limitations. KOA is a degenerative joint disease characterized by articular cartilage degeneration, synovitis, and osteophyte formation. Its pathogenesis is closely related to mechanical stress imbalance, abnormal elevation of proinflammatory factors (e.g., IL‐1*β* and TNF‐*α*), and chondrocyte metabolic dysfunction. TCM manual therapy of KOA is to regulate the metabolism of articular cartilage and the production of inflammatory factors through the release of local soft tissue of the knee joint and supplemented by joint motion manipulation. Clinically, KOA should be treated from the perspective of syndrome differentiation, combined with modern anatomy and biomechanics, and pay attention to the circulation of the channel tendons and restore the relative stability of the human structure. In addition, manipulation combined with relevant Chinese medicine therapies such as fumigation, acupoint injection, acupuncture, and so on. can effectively relieve KOA in the elderly. However, there are still some problems in the treatment of KOA by TCM manipulation: for example, the pathogenesis of KOA is not clear, and the mechanism of action of TCM manipulation in the treatment of KOA is still under study. Whether it is the use of holistic, local, or comprehensive therapy, it is still necessary to further clarify its principle of action and better guide clinical practice.

## 1. Introduction

**Figure 1:**
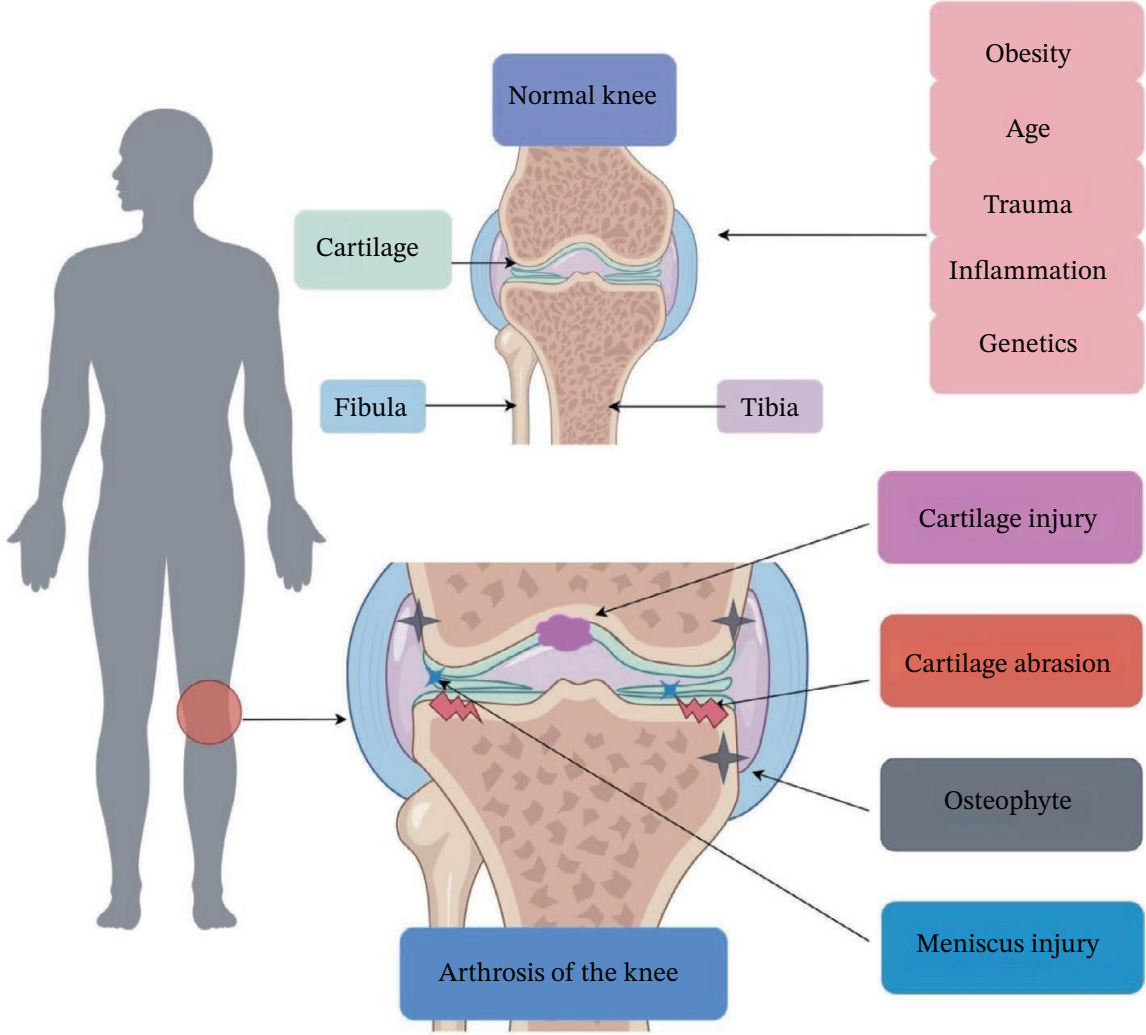
The influence factors of knee osteoarthritis occurred.

Knee osteoarthritis (KOA) is one of the most prevalent diseases in middle‐aged and elderly people, with clinical manifestations of knee pain, swelling, stiffness, activity limitation, [1, 2] and even psychological problems [3], which seriously affects the survival quality of patients, and the pain is highly correlated with their own function. Its pathogenesis is still under study, and it is mainly believed that the pathogenic factors alter the biomechanics of the knee joint or the molecular biology of the chondrocytes, extracellular matrix and subchondral bone [4, 5] to produce damage and destruction, and the equilibrium relationship is disordered. Related studies have found that obesity, age, trauma history, and genetic polymorphisms are risk factors for the development of KOA in the elderly [6], as shown in Figure 1 (By Figdraw). Some studies have shown that synovial inflammation, degeneration of cartilage and subchondral bone, and joint inflammation are important causes of KOA development. Some studies have confirmed that synovial inflammation correlates with KOA imaging, and imaging can be used clinically as one of the evaluation indicators for the degree of KOA lesions [7]. Nonsurgical treatments [8], especially traditional Chinese medicine (TCM) bone‐setting techniques, are favored by patients. In this article, we review the biological mechanism of Chinese manipulation treatment of KOA, the theoretical guidance of TCM, the related effects and combination therapy.

## 2. Methods

### 2.1. Literature Search

We searched the following databases for articles published from the inception of each database to May 2025, with the language restricted to English or Chinese. An updated search was conducted in February 2026. The databases searched include Wanfang Database, China Science and Technology Journal Database (VIP Database), China National Knowledge Infrastructure (CNKI), and PubMed. All keywords were mapped to “indexed items” (such as Medical Subject Headings [MeSH]) using the following combinations:

1. “knee”/exp (expanded subject heading for “knee”).
2. knee: title, abstract, keywords.
3. [<1966–2025]/publication year.
4. (#1 OR #2) AND #3.
5. “arthritis”/exp (expanded subject heading for “arthritis”) OR “osteoarthritis”/exp (expanded subject heading for “osteoarthritis”).
6. arthrit: title, abstract, keywords OR osteoarthr: title, abstract, keywords.
7. [<1966–2023]/publication year.
8. (#5 OR #6) AND #7.
9. “bone‐setting manipulation”/exp (expanded subject heading for “osteopathic manipulation”) OR “musculoskeletal manipulation”/exp (expanded subject heading for “musculoskeletal manipulation”).
10. massage: title, abstract, keywords OR “zone therap” (zone therapy): title, abstract, keywords OR manipul: title, abstract, keywords.
11. [<1966–2025]/publication year.
12. (#9 OR #10) AND #11.
13. “western ontario and mcmaster universities osteoarthritis index”/exp (expanded subject heading for “Western Ontario and McMaster Universities Osteoarthritis Index”) OR “visual analog scale”/exp (expanded subject heading for “Visual Analog Scale”)
14. “western ontario and mcmaster universities osteoarthritis index” OR womac
15. “visual analog scale” OR vas.
16. #13 OR #14 OR #15
17. “randomized controlled trial”/exp (expanded subject heading for “Randomized Controlled Trial”) OR “randomized controlled trial (topic)”/exp (expanded subject heading for “Randomized Controlled Trial (Topic)”) OR “controlled clinical trial”/exp (expanded subject heading for “Controlled Clinical Trial”) OR “randomization” (randomization).
18. (clinic NEAR/2 trial): title, abstract, keywords.
19. random: title, abstract, keywords OR placebo: title, abstract, keywords OR blind: title, abstract, keywords OR mask∗: title, abstract, keywords.
20. #17 OR (#18 AND #19).

### 2.2. Study Selection

Only randomized controlled trials (RCTs) that reported the use of a randomization method specifically for the application of bone‐setting manipulation alone in the treatment of KOA were included. For RCTs with three or more groups, if two of the groups met the inclusion criteria, the relevant literature was included. All patients with KOA were included in the study, regardless of their age, ethnicity, gender, age restrictions, or disease severity. Studies were excluded if they failed to report the randomization method, ethical approval status, or clinical trial registration information. Case reports, empirical reports, and laboratory studies were not included.

### 2.3. Inclusion Criteria

Patients included in the study were required to have a definitive diagnosis of KOA and meet other diagnostic criteria, such as those established by the American College of Rheumatology or the “Guidelines for the Diagnosis and Treatment of Osteoarthritis (2018 Edition)” by the Orthopaedic Branch of the Chinese Medical Doctor Association, with no restrictions on disease severity. The intervention in the experimental group consisted solely of bone‐setting manipulation therapy, whereas the intervention in the control group could be any other therapy besides manipulation therapy (e.g., acupuncture, medication, exercise therapy, and standard care). Additionally, studies that potentially included manipulation therapy as part of comprehensive care were included if the observed differences could be attributed to the unique effects of manipulation therapy, that is, studies where the effects of manipulation therapy could be isolated. For example, studies comparing bone‐setting manipulation therapy combined with standard care to standard care alone were included, whereas studies comparing bone‐setting manipulation therapy combined with standard care to manipulation therapy alone were excluded. We also excluded studies that combined bone‐setting manipulation therapy with other therapies, as it was difficult to distinguish the effects of bone‐setting manipulation therapy in such cases.

### 2.4. Outcome Analyses

The effects of bone‐setting manipulation combined with any other adjuvant therapies (including usual care, herbal external application, oral analgesics, exercise therapy, and acupuncture) were examined. The primary outcome measure was the Visual Analog Scale (VAS) for pain assessment. Secondary outcome measures included the pain score on the Western Ontario and McMaster Universities Osteoarthritis Index (WOMAC), follow‐up data, and adverse events.

### 2.5. Data Extraction

**Figure 2:**
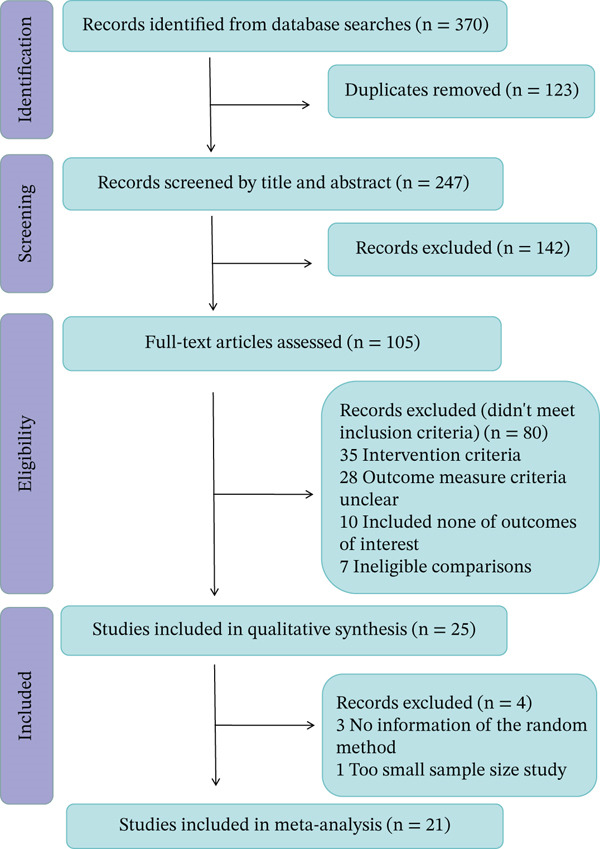
Selection of studies through review.

Data collection was independently conducted by two authors (Q.F. and X‐D.Z.), encompassing the following: number of subjects, gender, age, body mass index (BMI), disease duration, country, intervention period of bone‐setting manipulation, diagnostic criteria for KOA, Kellgren–Lawrence grade, VAS score, WOMAC pain score, follow‐up duration, and incidence of adverse events. In the meta‐analysis, VAS and/or WOMAC pain score data were extracted. If the data were insufficient, the corresponding authors were contacted to obtain supplementary data. The detailed process of the search and selection is shown in Figure 2.

### 2.6. Data Synthesis and Analysis

All search results were imported into NoteExpress v3.5.0 software for management. Two reviewers independently screened all potentially eligible studies. Initially, titles and abstracts were screened to exclude irrelevant literature. Subsequently, the full texts of all articles with potentially relevant abstracts were retrieved and screened according to the study inclusion criteria. Any disagreements were resolved through consensus or discussion with a third reviewer.

## 3. Biological Effects of Chinese Manipulation in the Treatment of KOA

### 3.1. Local Tissue and Joint Inflammation and Progressive Cartilage Loss and Bone Remodeling

It has been suggested that chondrocytes are needed to maintain the extracellular matrix of articular cartilage, and chondrocyte damage will lead to degeneration of articular cartilage. The pathological changes in articular cartilage tissue are closely related to the degradation of cartilage matrix by protein hydrolases; the extracellular matrix of cartilage is composed of collagen and proteoglycans, and so on, and proteoglycans are bound to polyproteoglycans outside the chondrocyte matrix through core protein chains, and osteoarthritis begins to occur in the early stage from the depletion of polyproteoglycans [9, 10]. Jin et al. analyzed the effect of Rijin Zhengbao manipulation on reducing the production and release of inflammatory factors and improving the synovial inflammation in KOA rabbits by inhibiting the activation of the TLR4‐MyD88‐NF‐*κ*B signaling mechanism. From the perspective of molecular biology, it is suggested that this manipulation may activate the body′s own regulatory mechanism through external stimulation, inhibit the excessive transmission of the TLR4‐mediated inflammatory signals, and then block the downstream signal amplification process that MyD88 depends on. The technique can inhibit TLR4‐mediated inflammatory signal overtransmission, block MyD88‐dependent downstream signal amplification, and ultimately regulate the activation of NF‐*κ*B to reduce the production and release of inflammatory factors, thus reducing the inflammatory response of KOA and improving the arthropathic lesions [11]. By analyzing the relevant lower limb mechanical indexes of full‐length X‐ray films of both lower limbs in the weight‐bearing position in 333 patients with KOA, Li et al. found that femoral and tibial rotations of the affected limbs were often present in patients with KOA, suggesting that the focus of Chinese medicine manipulative therapy should be focused on the reset of lower limb rotations [12]. In an experiment by [13], microcomputed tomography (micro‐CT) was used to measure articular cartilage thickness in the lateral femoral condyles of rabbits (*n* = 12 per group), randomized into an intervention group (receiving routine manipulation) and a control group (no intervention). The intervention group exhibited significantly slower cartilage thinning compared with controls (*p* < 0.05, two‐sample *t*‐test). Li [14] et al. collected synovial fluid from model rabbits (*n* = 15 per group) by aspiration from the joint and used an enzyme‐linked immunosorbent assay (ELISA) to quantify the levels of IL‐1 and MMP‐3 in the manipulation group and the model control group. The results showed significantly lower levels of IL‐1 (*p* = 0.012) and MMP‐3 (*p* = 0.035) in the intervention group, which suggests that manipulation improves blood circulation in the joint, increases lymphatic drainage, accelerates the metabolic clearance of inflammatory mediators, reduces the amount of MMPs in the joint cavity, and therefore preserves cartilage metabolism and reduces cartilage destruction.

### 3.2. Reduced Production of Inflammatory Factors

There is a large body of clinical evidence to suggest that inflammation plays a key role in the process of osteoarthritis of the knee and is one of the main causes of pain generation. Compared with inflammatory factors in serum [15], the level of inflammation in the synovial fluid of the knee joint is more likely to have a direct influence on the occurrence of inflammation and the metabolism and degradation of articular cartilage. Studies have suggested that interleukin‐1*β*(IL‐1*β*), tumor necrosis factor‐*α* (TNF‐*α*), and IL‐6 play a key role in and are associated with pain in the early stages of KOA [16]. Noyman et al.[17] found that there are a large number of adipocytokines in the bone mineral of KOA and that adiponectin and visfatin regulate cartilage cells, examining bone and adipokines. Increased release of proinflammatory factors, one of the causes of induced pain in KOA, is proinflammatory factors. Proinflammatory cytokines include IL‐1*β*, chemokines, and TNF‐*α*. IL‐1*β* is able to degrade portions of the extracellular matrix of joints, leading to deforming cartilage breakdown, whereas TNF‐*α* is an important mediator in cartilage matrix breakdown, which can make synovial tissue fibrosis worse in KOA patients, further aggravating KOA symptoms.

## 4. Theoretical Orientation of Chinese Medicine Manipulative Treatment for KOA

### 4.1. Balance of Muscles and Bones

According to TCM, the development of bone disease is closely related to the fullness of kidney qi, the kidney is the main bone to store the essence, and the essence produces the marrow; the liver is the main tendon to store the blood, and the essence of water and grain is used to nourish the meridians and channels. KOA belongs to the category of “paralysis,” “bone paralysis,” “knee paralysis,” and its pathogenesis belongs mostly to the internal deficiency of positive qi and deficiency of liver and kidney. KOA belongs to the category of “paralysis,” “bone paralysis,” “paralysis of the knee,” and its disease is localized in the joints of muscles and bones, and its disease mechanism belongs mostly to the internal deficiency of positive qi and deficiency of liver and kidney. Most of the diseases of the joints of muscles and bones are related to chronic and infected efforts with external evils, the functioning of qi and blood is violated, and the stagnation of qi and stagnation of blood is retained in the joints. Doctor Zhang′s guide to pain: “There is no knee pain that is not due to a deficiency of the liver and kidneys.” In treatment, the deficiency samples mainly come from the liver, spleen and kidneys, and the deficiency is supplemented to strengthen the muscles and bones; whereas the real samples consist of activating blood circulation and removing blood stasis, dispelling cold and dampness, and dredging meridians and collaterals. Yellow Emperor′s Internal Canon, medical text c. Three hundred BC: “All tendons belong to joints,” the early stage of tendon disease is mostly caused by the imbalance of “tendons” and various “tendon stagnations,” and the early stage of tendon injury is mostly corrected by muscles, tendons, contractures, misalignments, and locked tendons. Tendon contraction, misalignment, blockage from the beginning, to restore the balance of the tendon by manipulating the tendon; tendon and can bind the bone, tendon disease is not healed or wrong treatment of the first bone injury, then there is a certain degree of “bone error” in the treatment of the tendon at the same time to pay attention to changes in the condition of the bone, the tendon is smooth, the bone is correct; tendon disease is prolonged for a long time and bone and tendon and bone injuries with the same disease, it is simply tendon manipulation techniques difficult to maintain true balance, must complete the class joint manipulation movement to change the state of “bone error,” to restore the balance of tendon, bone and stability [18].

### 4.2. The General Principles of the Pain

The “Classic Evidence Treatment and Cure—Paralysis” describes that wind, cold and dampness attack, liver and kidney qi insufficiency and blood stagnation block the meridians, qi and blood, resulting in a prolonged and intractable course of the disease and joint pain. It has been found that the state of blood stasis is closely related to the inflammatory response, and worsening inflammation can lead to an imbalance of inflammatory/anti‐inflammatory cytokines [19], resulting in increased capillary permeability, damage to vascular endothelial cells, hemodynamic disturbances, coagulation abnormalities and the appearance of blood stasis. The abnormal state of blood stasis further aggravates the inflammatory response [20], the release of inflammatory factors increases [21] and inflammation worsens. In the treatment of musculoskeletal conditions, palpation to find knots in tendons and pressure points promotes blood circulation around joints, improves local tension and increases joint mobility, thus improving pain, stiffness and local function in patients with OAJ [22]. Wan et al. [23] conducted a RCT on 60 patients with KOA, which were divided into a TCM group (with the use of the elimination of stasis and bone connecting powder) and a western medicine control group (glucosamine). Through the coagulation indexes (prothrombin time [TT], prothrombinogen time [PT], partial thromboplastin time [APTT], fibrinogen [FBG], fibrinogen degradation product [FDP], international normalized ratio [INR], D‐dimer [D‐D]) tests, it was found that compared with the pretreatment period, the observation group′s TT, FBG, and FDP were significantly lower after treatment (*p* < 0.05 or *p* < 0.01); TT was significantly lower in the control group (*p* < 0.05). Compared with the control group after treatment, FDP was significantly lower in the observation group (*p* < 0.05). In addition, Il‐35 mRNA expression was elevated and Il‐12 mRNA was reduced in the group of eliminating blood stasis and bone connecting powder compared with that before treatment, and IL‐12 secretion was reduced in the group of eliminating blood stasis and bone connecting powder. According to the Yellow Emperor′s Canon Internal Medicine, “paralysis in the bones is heavy, in the veins it is blood clotting and in the tendons it is unfavourable for flexion and extension,” so Chinese doctors treat KOA by relaxing the tendons and activating the collaterals and regulating qi and blood.

## 5. The Role of Different String Management Maneuvers on the KOA

### 5.1. Different Tendon Management Maneuvers

The effectiveness of short‐term manipulation on pain is clear and widely used in clinical practice. Huang Jun‐ming et al.[24] evaluated the effect of Jianxiang Rigid Tendon Manipulation on the typing and staging treatment of KOA by a RCT. One hundred twenty patients were included and randomly divided into the intervention group (patellofemoral, tibiofemoral, and mixed types of typing manipulation treatment) and the control group (general Tui Na manipulation treatment), and were treated once on every other day, with a total of 10 treatments for a total of two treatments. The results showed that the VAS pain score of the intervention group was significantly lower than that of the control group (2.10 ± 0.30 vs. 2.90 ± 0.50, *p* < 0.01, ANOVA analysis), and the total effective rate was higher (86% vs. 68.75%, *p* < 0.01). This study suggests that manipulative therapy should be scientifically adjusted in terms of “formality,” “intensity,” “concentration,” and “total amount”. Li and Fan [25] confirmed the concepts of tendon‐bone dependence and tendon‐bone mechanical imbalance in Pengel′s theory of osteopathic manipulation by analyzing the clinical observation of Pengel′s osteopathic manipulation in the treatment of KOA, focusing on the correction of manipulation, and the correction of dysfunctional joints during treatment, so as to relieve pain and restore joint mobility. Sixty‐eight patients with KOA were included in the study, and the levels of blood inflammatory factors before and after the manipulation treatment were evaluated using the laboratory index test IL‐1 level. The results showed a significant reduction in IL‐1 levels in the treatment group after the manipulation (posttreatment difference in the intervention group: 72.22 ± 10.31 vs. 154.13 ± 10.01 before treatment, *p* < 0.01). This study emphasizes that manipulation relieves pain and improves function by “correcting joint misalignment” and “restoring mechanical balance”. Li et al. [26] used Professor Liang Zujian′s technique of “pressing, kneading and shaking” in the treatment of KOA, and included 88 patients who were randomly divided into the intervention group (manipulation + sodium vitrate injection) and the control group (sodium vitrate injection). In the intervention group, the Lysholm knee scoring scale (LKSS) score was 84.50 ± 5.05 vs. 78.11 ± 5.62 in the control group (*p* < 0.01), and serum TNF‐*α* (4.45 ± 1.31 *μ*g/L vs. 5.24 ± 1.41 *μ*g/L, *p* < 0.01) and MMP‐*α* (4.45 ± 1.31 *μ*g/L vs. 5.24 ± 1.41 *μ*g/L, *p* < 0.01) were found in the KOA patients after treatment. 0.01) and MMP‐9 (17.16 ± 3.41 *μ*g/L vs. 21.21 ± 4.23 *μ*g/L, *p* < 0.01) levels in serum were significantly reduced, suggesting that the maneuver reduces the release of TNF‐*α* and so on by inhibiting the relevant pathways, thus improving local microcirculation and tissue repair. Chen et al. analyzed [27] compared 38 patients with KOA and 38 normal patients with lumbar‐pelvic sagittal plane‐related parameters (standing lumbar spine lateral radiographs, measurement of lumbar lordosis (LL), pelvic projection (PI), sacral tilt (SS), pelvic tilt (PT), and independent samples *t*‐test to analyze the differences between the sagittal plane parameters between the groups), and found that the patients with KOA had a spine‐pelvic sagittal plane imbalance was found in patients with KOA, and the angle of knee flexion deformity was positively correlated with lumbar spine degenerative disease. Li Xi et al.[28] analyzed the hip push‐shake‐pull‐extension method for the treatment of initial and early KOA, and included 148 patients randomly divided into an intervention group (using the hip push‐shake‐pull‐extension method on the basis of health education and treated once every 2 days) and a control group (treated with oral diclofenac sodium extended‐release tablets on the basis of health education and treated with 75 mg/d). The maneuver combines the holistic concept of Chinese medicine with modern biomechanical knowledge, and the preliminary evidence suggests that the maneuver may reduce the abnormal loading of the knee joint by adjusting the relative position of the femoral head‐acetabulum and restoring the line of force of the lower limb. Sun et al. [29] explored the application of intervention component analysis using Tui Na treatment for KOA as an example, and found that treatment site, treatment purpose, and applied treatment are the main elements that affect the effectiveness of treatment, and that treatment intensity, changes in patient condition, and posttreatment risk factors in the treatment group are the main elements in maintaining treatment effectiveness and ensuring safety. They are of guiding importance for the design of subsequent clinical diagnosis and treatment programs. Wei′s trauma technique loosens adhesions, improves microcirculation, and enhances the body′s repair ability by pointing, kneading, pushing, and squeezing the internal and external pain points of the knee, thereby restoring joint stability [30] improving pain and other symptoms, and relieving meniscus degeneration [31]. Zhao Mingyu′s group applied the technique of “tendon stagnation and bone error” and proposed the diagnosis and treatment concept of “tendon as first, balance as use,” which attaches importance to the balanced relationship between the tendon and the inside and outside of the knee joint, thereby guiding the treatment of knee joint lesions and relieving the pain of tendon and bone diseases [32–34]. Zhao Mingyu′s group applied the technique of “tendon stagnation and bone error” and proposed the diagnosis and treatment concept of “tendon as first, balance as use.”

### 5.2. Holistic Dialectics

One of the core of the Chinese medicine system is the concept of the whole body, the human body, is an organic whole, the normal circulation of blood and fluids that people that peace and when any part of the injuries, will lead a hair moving the whole body. Hip and knee in “Medical Zong Jinjian”: “popliteal within the tendon, on the lumbar and crotch ……” explains that when the knee joint occurs when the wrong bone seam and other dislocations, the tendon connects the bone, the tendon ligament properly pulling, this will cause the patient to have a bend, hip flexion when the lumbar and hip pain. Jia et al. [35] of 78 cases of patients with cold‐wet paralytic obstruction type KOA, the use of lumbar‐hip‐knee coadjustment maneuver combined with warm paralysis and tendon shuzhi TCM topical comparing warm paralysis and tendon shuzhi TCM topical compression to adjust the lower limb line of force, corrects joint misalignment, the whole mainly taking care of local treatment of cold‐wet paralysis and KOA‐type obstruction, and restoring the balance condition of tendons and bones. In addition, in the clinical observation, it was found that VAS pain scores decreased by 0.6‐1.4 points (*p* < 0.05) and joint mobility increased, indicating that the overall regulation helped to restore the balance of the tendons and bones and improve the pathological state. Waist–hip–knee is a mechanical assembly, coregulation of the balance of the body, external evil attack on the human body, tendons and bones from the normal state of physiological balance breaks down, appearing bone is not firm, tendons are not Wing, long time stagnation of tendons and wrong bone appear “wrong bone seam, tendon in the groove” [36]. This is consistent with modern biomechanical studies of tissue strain and load transfer anomalies. Professor Zhang Zhenyu [37] believes that the local knee joint injury makes the hip, spine and abdomen function change, and the change of hip, spine and abdomen function makes the knee joint pain and flexion and extension function decrease, local and global cause and effect. The treatment adopts eight parts to adjust the knee, three steps to adjust the hip, three methods of spine and abdomen, four in one, multicausal adjustment to reduce pain, promote qi and blood function, enhance lower limb muscle strength and independent exercise, early intervention, early treatment. Zhang et al. [38] explored the feasibility of holistic “waist‐hip‐knee‐ankle” massage therapy for KOA from the perspective of meridian theory, myofascial theory and lower limb biomechanics, which reflects the holistic view of Chinese medicine. Manipulation helps to slow down degenerative changes by improving stress distribution in the joints and reducing the soft tissue stress response. Liu et al. [39] used mate analysis to systematically evaluate the efficacy and safety of holistic osteopathic manipulation in the treatment of KOA and found that holistic osteopathic manipulation effectively relieved knee pain and stiffness and improved knee mobility. Pan Fuwei et al. [40] started from the theory of “tendon and bone imbalance” and used biomechanical techniques to observe biomechanical changes in the lower limbs of KOA patients when walking, and analyzed muscle and bone changes, that is, tendon and bone changes, in terms of kinetics, dynamics and kinematics.

## 6. Manipulation in Combination With Other Therapies for KOA

### 6.1. Manipulation in Combination With Warm Acupuncture and Moxibustion

Wang et al. [41] used the techniques take, click, push with one finger, flexion and extension of the joints along with warm acupuncture and moxibustion on the inner knee eye, outer knee eye, Yanglingquan, Yinlingquan, Liangqiu, and Sanli foot. Warm acupuncture and moxibustion combined with acupressure can effectively improve patients′ symptoms, suppress inflammatory stimulation, reduce IL‐1, TNF‐*α* and MMP‐3 levels, suppress bone resorption, and promote bone production, and the clinical effect is accurate.

### 6.2. Manipulation Combined With Fumigation With TCM

Reduce the level of inflammatory factors. Liu et al. [42] analyzed the clinical efficacy of massage combined with fumigation with TCM in the treatment group and the control group combined with oral gucci gel capsule and topical ointment. Liu et al. [43] found that manipulation to establish Pingol′s bone combined with oral administration of fascial pain elimination pills and herbal fumigation could improve the stiffness of surrounding muscles, relieve pain, and improve knee flexion and extension.

### 6.3. Manipulations Combined With Oral Administration of TCMs

TCM believes that most of the tendon and bone problems of the human body are related to the liver and kidneys and the invasion of external evil, so the treatment choice of TCM is mainly based on tonifying the liver and kidneys and dissipating the evil. Xu et al. [44] enrolled 137 patients diagnosed with liver and kidney insufficiency, phlegm and blood stasis in their study and randomly grouped them into one group. The treatment group received oral treatment with Dispelling Paralysis and Nourishing Knee Formula along with three‐step and nine‐step knee manipulation, whereas the control group received oral treatment with celecoxib and glucosamine capsules along with a low‐frequency physiotherapy instrument. Dispelling Paralysis and Nourishing Knee Formula combined with three‐ and nine‐step knee manipulation were found to be effective in the treatment of mid‐ to early‐stage KOA with hepatic and renal insufficiency, phlegm and blood stasis in bone. Hong et al. [45] analyzed the short‐term efficacy of rotational bone fixation manipulation combined with Wenyang Tongluo capsules in the treatment of Yang deficiency and cold coagulation‐type KOA and found that the therapy could improve efficacy and reduce patient anxiety.

### 6.4. Manipulation Combined With Exercise Therapy

Lou et al. [46] found that exercise promotes angiogenesis. Therefore, exercise therapy is essential in KOA treatment to avoid daily use. Exercise therapy includes self‐muscle exercises and traditional gong methods. Taijiquan, Yi Jin Jing, Ba Duan Jin, and Wu Bird Opera are among the traditional gong methods. Nigam [47] studied 40 patients with KOA and compared exercise in combination with conventional treatment with conventional treatment alone, and there was significant improvement in patients′ pain and joint mobility. Hu et al. [48] used manipulation with exercise therapy (quadriceps and flexor muscle contraction training) and wearing knee pads in combination with static and dynamic therapy to effectively exercise the quadriceps and other flexor and extensor muscles around the knee joint, reduce muscle inhibition, increase muscle strength of the quadriceps and surrounding flexor and extensor muscle groups, improve joint stability, and effectively strengthen the role of the “tendon bundle bone.” The role of the “tendon bundle bone” is effectively strengthened. Active exercise can also accelerate venous return, reduce the accumulation of inflammatory mediators, reduce local irritation, and thus reduce pain. Wearing a knee brace for relative fixation of the knee joint can support joint force from outside the joint while limiting stimulating activities such as knee flexion and hyperextension to increase joint stability. Li et al. [49] found that Eight‐Duanjin exercises with electroacupuncture and massage for the treatment of KOA in the middle and early stages of the disease were able to improve pain threshold, joint stability, and joint mobility, improve patients′ quality of life, and reduce the recurrence rate. Fang Min et al. [50] observed and compared the different effects of Yi Jin Jing gongfu and stretching exercise on the biomechanical parameters of gait in patients with KOA, and gongfu exercise was able to increase knee flexion and extension moments, reduce intra‐articular retraction moments and other kinematic parameters of the lower limbs in KOA, and increase the range of motion of the knee joint. Song et al. [51] analyzed the effect of modified Tai Chi exercises on lower limb muscle strength and cardiorespiratory endurance before and after treatment of KOA in elderly women and found that patients′ quadriceps muscle strength and fatigue resistance improved, active and antagonist muscle coordination improved, and strength control and stability improved. Ferreira et al. [52] concluded in a systematic review and meta‐analysis that nonpharmacological and nonsurgical interventions were effective in the treatment of KOA. Ferreira et al. [52] concluded through a systematic review and meta‐analysis that exercise therapy is effective in patients with KOA and that pulsed electromagnetic fields and moxibustion are also good treatments.

### 6.5. Manipulation combined with acupuncture injections

Luo Caigui et al. [53] used the four steps of the Emei Injury Research School′s Tui Na (“Loose, divided, warm, smooth “) in combination with acupuncture injection of mesenchymal stem cells to treat KOA, and evaluated knee joint MRI before and after treatment and found that the treatment was able to effectively promote joint recovery and improve patients′ clinical symptoms.

### 6.6. Manipulation Combined With Acupuncture

Wang et al. [54] observed the treatment of KOA in the elderly by acupuncture “relative points” Yinlingquan and Yanglingquan with seven‐step massage, and the mobility and biomechanics of the knee joint of the patients improved, and the prognosis of the patients was good.

### 6.7. Manipulation Combined With External Application of Plaster

Dong et al. [55] observed the clinical study of “soft tendon and core nutrition” manipulation with cream removal on the early and middle stage of KOA, and found that it can loosen the “knot” and “pool” points of the meridional tendon “pool,” so that the local pressure of the joints can rebuild their balance, and the local pressure of the joints can be reduced and the balance of the joints can be improved. By loosening the “knots” and “clusters” of the meridian tendons, it can rebalance the local stress of the joints, moisturize and nourish the meridian tendons, reduce the pressure in the joint cavity, and relieve adhesions around the joints.

## 7. Critical Appraisal of Methodological and Statistical Reporting in Included Studies

Although the majority of studies reviewed in this article (e.g., those cited in Sections 3.1, 5.1, and 6.1–6.7) report positive outcomes, they exhibit significant heterogeneity in their statistical reporting. For instance, some studies employed only *t*‐tests or chi‐square tests without verifying whether the assumptions for these parametric tests were met. More critically, most studies reported only *p* values, lacking essential estimates of effect size (e.g., Cohen′s d, standardized mean difference) and 95% confidence intervals. This omission makes it difficult to judge the clinical significance and precision of the reported findings. Furthermore, there is considerable variability in the outcome measures used across studies (e.g., VAS pain scores, WOMAC index, Lysholm score, range of motion, and inflammatory factor levels), along with inconsistent timing of assessments (e.g., immediately posttreatment, 1 week, 1 month). This heterogeneity in endpoints and measurement schedules presents a major obstacle for quantitative synthesis (e.g., meta‐analysis) and hinders the ability to draw definitive conclusions. The limitations of clinical observations showing short‐term improvement must be acknowledged. Most trials on manipulative therapy face challenges in implementing perfect blinding. Additionally, the control conditions vary widely (e.g., wait‐list, usual care, and sham manipulation), and these methodological differences directly impact the comparability of results and the risk of bias. Future research should adhere to reporting guidelines such as CONSORT. It is imperative to mandate the reporting of effect sizes with confidence intervals, specify statistical tests used, and detail procedures for randomization and blinding. This will enhance the transparency, reproducibility, and overall quality of the evidence base.

## 8. Discussion

Unlike previous reviews focusing solely on efficacy or isolated mechanisms, this review systematically constructs a comprehensive “theory‐mechanism‐technique‐integration” framework for Chinese manipulation in KOA, spanning biological effects (Chapter 1), theoretical principles (Chapter 2), specific techniques (Chapter 3), to combination therapies (Chapter 4). It highlights the guiding role of the “muscle‐bone balance” theory and holistic dialectics in clinical application, offering a crucial theoretical complement to Western reviews emphasizing biomechanics. For treatment strategy, evidence suggests promising short‐term outcomes from combining manipulation with warm therapies (6.1, 6.2) or exercise therapy (6.4). Operationally, the primary short‐term goal should be muscle relaxation and improved joint mobility, guided by individual “muscle‐bone balance” assessment. Crucially, clinicians must be aware that current evidence, limited by methodological heterogeneity (as noted in Section 7), supports short‐term symptom improvement but cannot conclusively confirm long‐term superiority over other interventions. Future research should: (1) Elucidate mechanisms via longitudinal studies linking dynamic changes in biomarkers (e.g., CTX‐II, IL‐6) to imaging outcomes; (2) conduct high‐quality RCTs with large samples, long‐term follow‐up, standardized protocols, and rigorous placebo controls; (3) resolve the tension between developing standardized research protocols and preserving individualized holistic dialectical approaches.

Chinese manipulation is a valuable nonpharmacological option for KOA with a theoretical and preliminary practical foundation. However, the strength of evidence is constrained by methodological and reporting inconsistencies. Advancing the field requires more rigorous scientific methods and reporting standards to establish robust evidence for clinical decision‐making.

### 8.1. Ethical Oversight and Reporting Limitations

Most of the included clinical studies on Chinese manipulation for KOA have not explicitly reported detailed ethical oversight or approval information, which is a common limitation in the existing literature. Ethical approval is crucial to ensure the safety, rights, and wellbeing of participants, particularly when invasive procedures or novel interventions are involved. The absence of clear ethical reporting may reflect publication standards at the time of study or regional differences in ethical regulation.

Subsequent research endeavors should place primary emphasis on rigorous ethical review protocols and adhere to standardized transparent reporting frameworks in compliance with internationally recognized guidelines, including the Declaration of Helsinki, as well as the regulatory requirements stipulated by local Institutional Review Boards (IRBs). The lack of documented ethical oversight in a subset of the included studies may potentially undermine the external validity (generalizability) and scientific credibility of their reported findings. Accordingly, readers and clinical practitioners are advised to adopt a prudent approach when interpreting the efficacy and safety outcomes derived from these investigations.

## Author Contributions

Qiao Fan and Xiang‐Dong Zhang contributed equally to this work.

## Funding

The study was supported by the Henan Province Chinese Medicine Top Talent Cultivation Project (2019ZYBJ23); Institute of Basic Theory of Traditional Chinese Medicine, China Academy of Chinese Medical Sciences (YZ‐1883); Scientific Research Project of Traditional Chinese Medicine in Henan Province (2023ZY1021); Henan Medical Science and Technology Research Program (No. LHGJ20240452); Clinical effect of Pingle tendon stagnation bone malocclusion manipulation combined with Jinju analgesic gel in the treatment of knee osteoarthritis of kidney deficiency and blood stasis type in the early stage (No.2023GBJC03).

## Conflicts of Interest

The authors declare no conflicts of interest.

## Data Availability

Data sharing is not applicable to this article as no datasets were generated or analyzed during the current study.
